# RNF12 is regulated by AKT phosphorylation and promotes TGF-β driven breast cancer metastasis

**DOI:** 10.1038/s41419-021-04493-y

**Published:** 2022-01-10

**Authors:** Yongsheng Huang, Sijia Liu, Mengjie Shan, Sophie C. Hagenaars, Wilma E. Mesker, Danielle Cohen, Lin Wang, Zhi Zheng, Peter Devilee, Rob A. E. M. Tollenaar, Zhangfu Li, Yongmei Song, Long Zhang, Dan Li, Peter ten Dijke

**Affiliations:** 1 Institute of Basic Medical Sciences and School of Basic Medicine, Chinese Academy of Medical Sciences and Peking Union Medical College, Beijing, China; 2 Department of Cell and Chemical Biology, Leiden University Medical Center, Leiden, The Netherlands; 3 Department of Surgery, Leiden University Medical Centre, Leiden, The Netherlands; 4 Department of Pathology, Leiden University Medical Center, Leiden, The Netherlands; 5 Department of Human Genetics, Leiden University Medical Center, Leiden, The Netherlands; 6 Key Laboratory of Cancer and Microbiome, State Key Laboratory of Molecular Oncology, National Cancer Center/Cancer Hospital, Chinese Academy of Medical Sciences and Peking Union Medical College, Beijing, China; 7 Life Sciences Institute, Zhejiang University, Hangzhou, Zhejiang China

**Keywords:** Cancer, Cell biology

## Abstract

Transforming growth factor-β (TGF-β) acts as a pro-metastatic factor in advanced breast cancer. RNF12, an E3 ubiquitin ligase, stimulates TGF-β signaling by binding to the inhibitory SMAD7 and inducing its proteasomal degradation. How RNF12 activity is regulated and its exact role in cancer is incompletely understood. Here we report that RNF12 was overexpressed in invasive breast cancers and its high expression correlated with poor prognosis. RNF12 promoted breast cancer cell migration, invasion, and experimental metastasis in zebrafish and murine xenograft models. RNF12 levels were positively associated with the phosphorylated AKT/protein kinase B (PKB) levels, and both displayed significant higher levels in the basal-like subtype compared with the levels in luminal-like subtype of breast cancer cells. Mechanistically, AKT-mediated phosphorylation induced the nuclear localization of RNF12, maintained its stability, and accelerated the degradation of SMAD7 mediated by RNF12. Furthermore, we demonstrated that RNF12 and AKT cooperated functionally in breast cancer cell migration. Notably, RNF12 expression strongly correlated with both phosphorylated AKT and phosphorylated SMAD2 levels in breast cancer tissues. Thus, our results uncovered RNF12 as an important determinant in the crosstalk between the TGF-β and AKT signaling pathways during breast cancer progression.

## Introduction

Transforming growth factor-β, (TGF-β), plays a biphasic role in breast cancer progression by acting as a tumor suppressor during the early stages of cancer progression, and a tumor promoter during the late stages [1–3]. Although TGF-β elicits cytostatic responses in normal and pre-malignant breast cells, such responses are abrogated in malignant breast cells, wherein TGF-β stimulates the migration, invasion, and metastasis of malignant cells. TGF-β induces cellular responses by activating heteromeric complexes of TGF-β type I (TβRI) and type II (TβRII) receptors, respectively [4]. Upon TβRI phosphorylation by TβRII kinase, activated TβRI induces the phosphorylation of receptor-regulated (R)-SMAD2 and SMAD3, which form heteromeric complexes with the common mediator, SMAD4. These heteromeric SMAD complexes mediate transcriptional responses in collaboration with other DNA-binding transcription factors [5]. In breast cancer, mutations that inactivate TGF-β receptors and SMADs are not commonly found. Instead, TGF-β/SMAD-induced growth inhibitory effects are diminished or redirected via the activation of oncogenes [6]. One such pathway is the phosphatidylinositol kinase (PI3K)/AKT pathway, which is strongly activated by growth factors such as the insulin growth factor (IGF), and mediates cellular survival [6, 7]. An intricate interplay occurs between TGF-β/SMAD and PI3K/AKT pathways [8]. For example, AKT can sequester R-SMADs in the cytoplasm, thereby inhibiting TGF-β-induced apoptosis [9, 10] and both pathways also enforce each other to promote the invasion of breast cancer cells [7, 11].

Inhibitory SMAD7 is an important negative regulator of the TGF-β/SMAD pathway. It is rapidly induced by TGF-β, to participate in a negative feedback loop [12]. SMAD7 antagonizes TGF-β family signaling by recruiting SMURF E3 ubiquitin ligases to activated receptors and targeting them for degradation [13, 14]. Recently, OTUD1 was identified as a deubiquitinase of SMAD7. OTUD1 acts as a tumor suppressor in breast cancer by mitigating TGF-β-induced pro-oncogenic responses by increasing SMAD7 levels [15]. ARKADIA (RNF111) [16] and RNF12 (RLIM) [11] have been identified as E3 ubiquitin ligases acting on SMAD7, which mediate ubiquitination and proteasomal degradation. These are negative regulators of SMAD7, and promote TGF-β/SMAD signaling [17]. ARKADIA is involved in the early stages of breast cancer metastasis [18].

RNF12/RLIM was originally identified as a protein that binds to LIM homeodomain (HD) proteins and targets a cofactor of LIM protein (CLIM) for degradation [19, 20]. CLIM and RNF12 have been identified as opposing regulators of estrogen receptor (ER)-dependent transcriptional activity in breast cancer [21]. RNF12 mitigates the transcriptional activity of LIM-HDs but promotes transcriptional activation of ER target genes, and may thereby contribute to the stimulation of breast cancer cell proliferation [21]. RNF12 increases the turnover of the telomeric protein, TTAGGG repeat binding factor 1 (TRF1). Depletion of RNF12 in HT1080 fibrosarcoma cells increases TRF1 levels and induces telomere shortening [22], thereby impairing cell growth. In addition, RNF12 negatively regulates c-Myc/MIZ1 transcriptional activity and inhibits hepatocellular carcinoma cell proliferation [23, 24]. Moreover, RNF12 is an X-encoded, dose-dependent inducer of X chromosome inactivation (XCI) in mouse embryonic stem cells (ESCs) [25, 26]. Subcellular distribution and function of RNF12 are regulated via Ser214 phosphorylation by Ser/Arg (SR)-rich splicing factor (SRSF) protein kinase (SRPK) [27, 28]. Together, the hitherto described data suggest a complex role for RNF12 in the regulation of multiple signaling pathways, where its role in cancer progression may be context-dependent. In this study, we show that RNF12 enhances the TGF-β signaling pathway and plays a role in breast cancer cell migration, invasion, and metastasis. Our findings may provide some insights for understanding the underlying mechanism for breast cancer metastasis.

## Results

### RNF12 is critical for metastatic and invasive traits in breast cancer

**Fig. 1:**
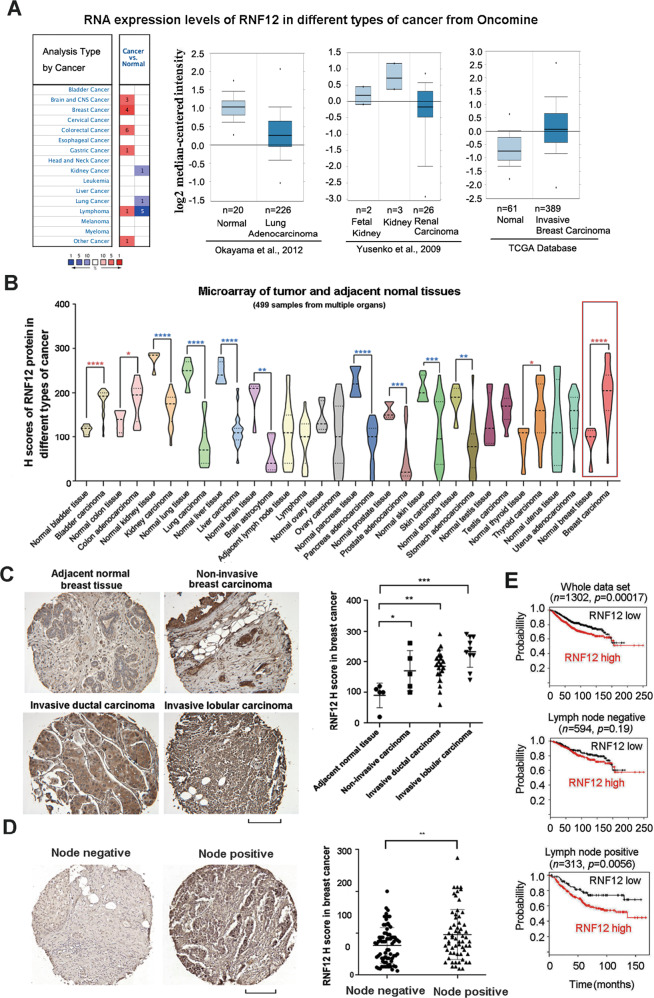
RNF12 expression in breast and other types of cancer. **A** Oncomine box plots of *RNF12* mRNA levels in different types of cancer from public datasets (www.oncomine.org). **B** Statistics of RNF12 protein levels expressed in 499 specimens of different cancer tissues compared with the matched adjacent normal tissues. The two-tailed Student’s *t*-test was used to determine the statistical significance (*p < 0.05; **p < 0.01; ***p < 0.001; ****p < 0.0001). **C** Representative images of RNF12 immunohistochemistry result in invasive breast cancer tissues and adjacent normal tissues. The intensity of RNF12 staining is shown in the right-hand panel. Scale bar = 300 μm. **D** Representative images of RNF12 immunohistochemistry results in lymph node-positive or lymph node-negative breast cancer tissues. The intensity of RNF12 staining in the 175 specimens of breast cancer (ORIGO) is shown in the right-hand panel. **E** RNF12 correlates with poor prognoses in lymph node-positive breast cancer patients. Kaplan–Meier curves (http://kmplot.com/analysis) show that metastasis-free survival of individuals was negatively correlated with RNF12 expression in lymph node-positive patients (bottom panel) but showed no correlation with RNF12 in lymph node-negative patients (middle panel).

To investigate the role of RNF12 in cancer progression, we analyzed the RNA expression levels of RNF12 in different cancer tissue samples in Oncomine. The RNA expression level of RNF12 was high in breast cancer compared with other types of cancer (Fig. 1A). Next, we determined the expression of RNF12 in a tissue microarray that included clinical samples from 16 different common cancers. RNF12 protein levels were nominally higher in breast, bladder, colon, thyroid cancer than in adjacent normal tissues (Fig. 1B), but the reverse was observed for kidney, lung, liver, brain, prostate, skin, pancreas and stomach cancer (Supplementary Fig. S1). Interestingly, further in-depth analysis of the 45 breast samples included on the array indicated that the expression level of RNF12 protein was high in invasive breast cancer, moderate in non-invasive breast cancer, and low in adjacent normal breast tissue (Fig. 1C). These results suggested that RNF12 may play a specific role in the invasiveness of breast cancer. To obtain further clinical evidence for this hypothesis, we examined RNF12 expression in another larger panel, which included 175 breast cancer tissue samples (ORIGO) [29]. We found that RNF12 expression was higher in lymph node-positive breast cancer samples than in lymph node-negative breast cancer samples (Fig. 1D). Moreover, Kaplan–Meier analysis of 1302 breast cancer patients from Kaplan–Meier plotter dataset (http://kmplot.com/analysis) showed that RNF12 amplification was associated with a poor clinical outcome in lymph node-positive breast cancer but not in the lymph node-negative form, suggesting that high RNF12 levels are associated with worse prognosis in lymph node-positive cancers. (Fig. 1E). Based on these results, we hypothesized that RNF12 drives breast cancer progression mainly by promoting invasive and metastatic traits.

### RNF12 regulates breast tumor progression in vivo

**Fig. 2:**
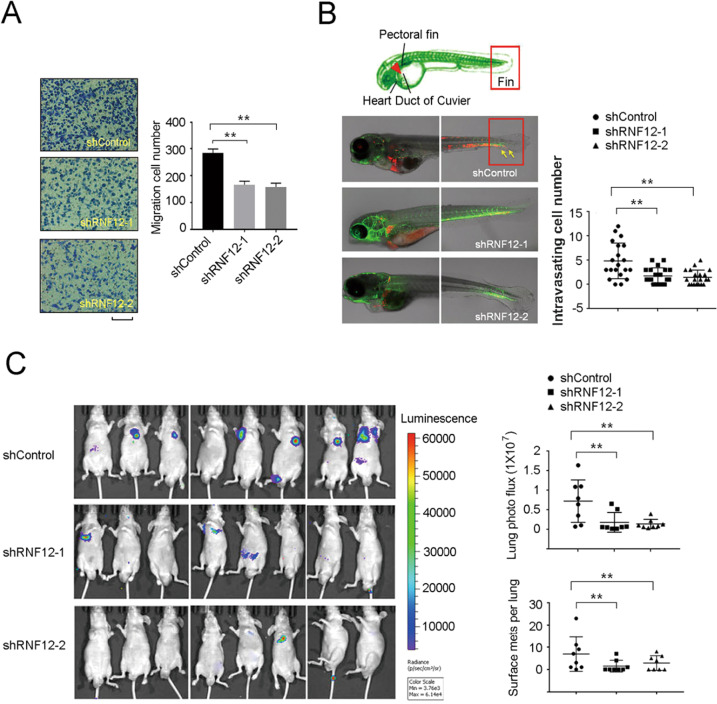
RNF12 increases invasion and metastasis of breast cancer cells. **A** Transwell migration of control or RNF12 stably depleted MDA-MB-231 cells. Left panel: Representative images of migrated cells. Right panel, fold changes of migrated cell numbers; mean ± SD of triplicates. Scale bar = 50 μm. **B** Schematic diagram of the zebrafish embryo; shRNF12 or control MDA-MB-231 cells were injected into the Duc of Cuvier of zebrafish embryos at 48 h post fertilization. Representative images of zebrafish at 5 dpi are shown. Percentage of zebrafish embryos exhibiting invasion at 5 dpi. The results of two independent experiments are shown. The yellow arrows indicate invasive cells. **C** Depletion of RNF12 inhibits lung metastasis of breast cancer in a transplantable nude mouse model. Left panel: representative bioluminescent images of mice injected with control or RNF12-depleted MDA-MB-231 cells into the tail vein 35 days after injection. Right panel: Bioluminescent signal and surface metastases in the lungs were quantified. Scatter plots are shown as mean ± SD. Statistical significance was set at *p* < 0.05 (*0.01 < *p* < 0.05; **0.001 < *p* < 0.01; ***p < 0.001).

To study the function of RNF12 in breast cancer progression, with particular reference to enhanced breast cancer migration and invasion, we silenced endogenous RNF12 expression in the highly aggressive basal MDA-MB-231 breast cancer cell line. We found depletion of RNF12 in MDA-MB-231 cells resulted in a significant decrease in cell invasiveness in the Transwell migration assay (Fig. 2A). Next, we tested the role of RNF12 in malignant MDA-MB-231 breast cancer cells in vivo using a previously published zebrafish embryo xenograft breast cancer model [8]. Zebrafish embryos were injected into the Duct of Cuvier (Doc) with mCherry-labeled MDA-MB-231 cells. Circulating cancer cells extravasate into the tail fin, which is enriched with collagen (Fig. 2B). Extravasation is a key step during the metastatic process. Embryos transplanted with RNF12-depleted MDA-MB-231 cells exhibited significantly less extravasation ability than embryos injected with control cells (Fig. 2B). Furthermore, we tested the role of RNF12 in an experimental breast cancer metastasis model using immunodeficient mice. RNF12-depleted MDA-MB-231 cells and control MDA-MB-231 cells were injected into the tail vein of female nude mice, respectively. We observed significant inhibition of MDA-MB-231 cells colonization in the lung when RNF12 expression was suppressed (Fig. 2C, Supplementary Fig. 2). We also constructed a stable MCF-7 cell line with ectopic expression of RNF12 (Supplementary Fig. 3A). We found that overexpression of RNF12 in MCF-7 cells can enhance the migration and invasion of MCF-7 cells (Supplementary Fig. 3B-C). Together, these results indicated that RNF12 promotes breast cancer cell migration, invasion, and metastasis in vitro and in vivo.

Furthermore, we analyzed the effect of *RNF12* depletion on the expression of TGF-β/SMAD-induced target genes that are important for the highly malignant and metastatic phenotype of MDA-MB-231 cells (Supplementary Fig. 4A-C). Spheroid assay indicated that *RNF12* depletion reduced the invasion ability of MDA-MB-231 cells induced by TGF-β signaling (Supplementary Fig. 4B). In addition, TGF-β-induced mRNA levels of various mesenchymal markers, including *SNAIL* [30], *SLUG* [30], *N-Cadherin* [28, 31], and direct TGF-β target genes *plasminogen activator-1* (*PAI-1)* [32]*, chemokine receptor CXCR4* [33]*, connective tissue growth factor (CTGF)* [34]*, matrix metalloproteinase 9 (MMP9)* [35]*, and vascular endothelial growth factor A (VEGF-A)* [36] were decreased in the cells following *RNF12* knockdown (Supplementary Fig. 4C). These results suggested that RNF12 depletion impairs the TGF-β/SMAD signaling response in MDA-MB-231 breast cancer cells, which is associated with mesenchymal as well as invasive/metastatic traits.

### AKT phosphorylates and stabilizes RNF12 protein

**Fig. 3:**
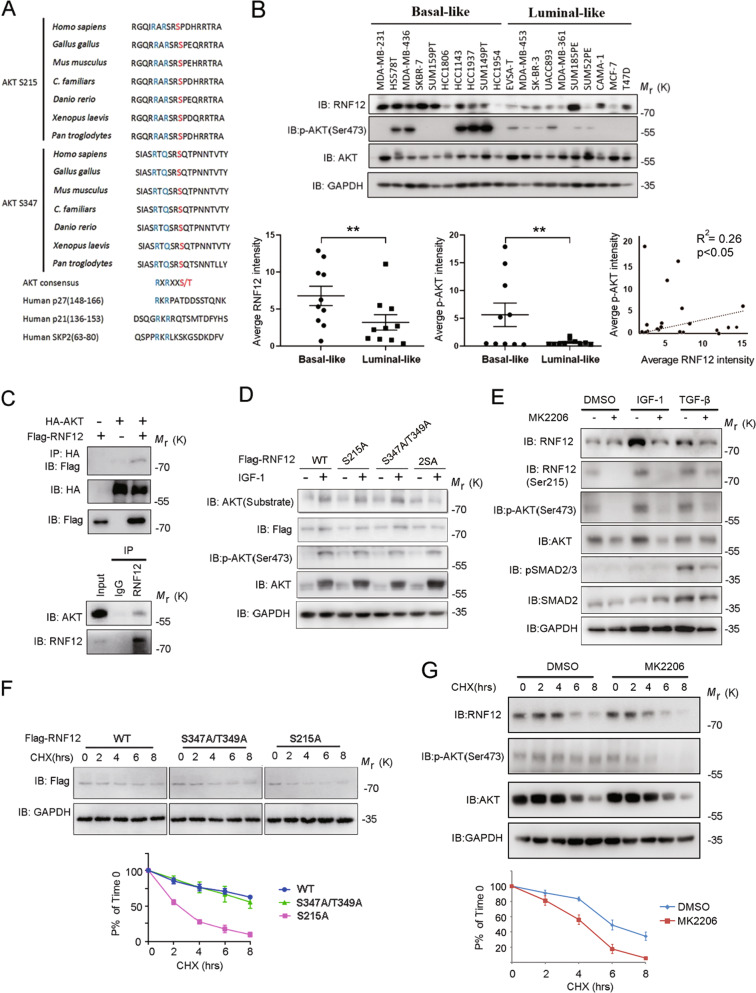
AKT phosphorylates and stabilizes RNF12 protein. **A** Sequence alignment of the AKT phosphorylation site within RNF12 orthologues of different species and known AKT substrates, p27, p21, and SKP2 **B** RNF12 protein was expressed at higher levels in basal-like breast cancer cell lines. Immunoblot analysis of RNF12 and pAKT expression in different breast cancer cell lines; GAPDH was used as the loading control. Scatterplot showing the positive correlation between pAKT and RNF12 expression in breast cancer cell lines (*0.01 < *p* < 0.05; **0.001 < *p* < 0.01; ***p < 0.001; Pearson’s coefficient tests were performed to assess significance). **C** Co-immunoprecipitation of Flag-RNF12 and HA-AKT transfection in HEK293T cells as indicated (left panel). Endogenous interaction between RNF12 and AKT was detected in MDA-MB-231 cells (right panel). Cell lysates were subjected to immunoprecipitation with RNF12 antibody, followed by immunoblotting with an AKT antibody. **D** AKT phosphorylates RNF12 at Ser215 and 347 in vitro. Flag-RNF12 mutants were transfected into 293T cells, and phosphorylation was detected using a phospho-AKT substrate antibody that recognizes either the RXRXXpS(pT) or the R-KXR-KXXpS(pT) motif. **E** Immunoblot analysis of whole-cell lysate derived from serum-starved MDA-MB-231 cells treated with IGF-1 (200 ng/mL), TGF-β (5 ng/mL), and MK2206 (5 μM) for 8 h, as indicated. **F** RNF12 S215A mutant affects protein stability. Top panel: Immunoblot analysis of HEK293T cells were transfected with the indicated plasmids and treated with CHX (20 μg/mL) for the indicated times. Bottom panel: Quantification of RNF12 band intensities. The band intensity was normalized to the time-zero controls. The results are represented by the mean ± SD of three independent sets of experiments. **G** AKT signaling affects endogenous RNF12 protein stability. Top panel: Immunoblot analysis of MDA-MB-231 cells treated with dimethylsulfoxide (DMSO) or MK2206 (5 μM) together with CHX (20 μg/mL) for the indicated times. Bottom panel: Quantification of RNF12 band intensities. The band intensity was normalized to the time-zero controls. The results are represented by the mean ± SD of three independent sets of experiments.

The above studies showed that RNF12 plays an important role in regulating TGF-β/SMAD signaling and breast cancer progression. To study the mechanism underlying the regulation of RNF12 activity, we first analyzed the RNF12 protein sequence. The primary amino acid sequence revealed that RNF12 contains two AKT consensus RxRxxS(T) phosphorylation motifs at Ser215 and Ser347, which are conserved in RNF12 orthologues (Fig. 3A). Next, we analyzed RNF12 protein levels in different breast cancer cell lines that we collected in previous study [37]. RNF12 was expressed at higher levels in the basal-like subtype than in the luminal-like subtype breast cancer cell lines (Fig. 3B). Interestingly, the expression levels of activated AKT (phosphorylated on Ser473; pAKT) in basal-like breast cancer cell lines were also significantly higher than that in luminal-like cell lines (Fig. 3B). Thereafter, we searched for a possible link between RNF12 levels and pAKT levels and observed a positive correlation (Fig. 3B).

Mechanistically, both exogenous and endogenous RNF12 interacted with the AKT protein (Fig. 3C). To examine whether RNF12 acts as a substrate for AKT, we used a phospho-specific antibody that recognizes the optimal AKT phosphorylation consensus motif. The AKT substrate antibody showed that IGF-1 induced phosphorylation of RNF12 wild-type (WT) protein in HEK 293 T cells, but no specific signal was detected with this antibody in the RNF12 double mutant proteins [2SA: RNF12 Ser215Ala (S215A) and Ser347Ala/Thr349Ala (S347A/T349A)] (Fig. 3D). Both IGF-1 and TGF-β induced phosphorylation of endogenous RNF12 was detected by the AKT substrate antibody (Fig. 3E). Notably, the protein level of RNF12 was significantly increased in cells treated with IGF-1, whereas this response was abrogated in cells treated with MK2206, a selective inhibitor of AKT [38] (Fig. 3E). Consistent with these findings, other results obtained from cycloheximide (CHX) pulse/chase experiments demonstrated that the half-life of RNF12 mutant S215A was shorter than that of RNF12 WT (Fig. 3F), while the stability of RNF12 S347A/T349A was comparable to that of RNF12 WT protein (Fig. 3F). In addition, we found the half-life of endogenous RNF12 protein was shorter when AKT activity was inhibited (Fig. 3G). These results suggested that AKT signaling stabilizes RNF12 protein mainly through the Ser215 site.

### AKT signaling is critical for the nuclear localization and function of RNF12

**Fig. 4:**
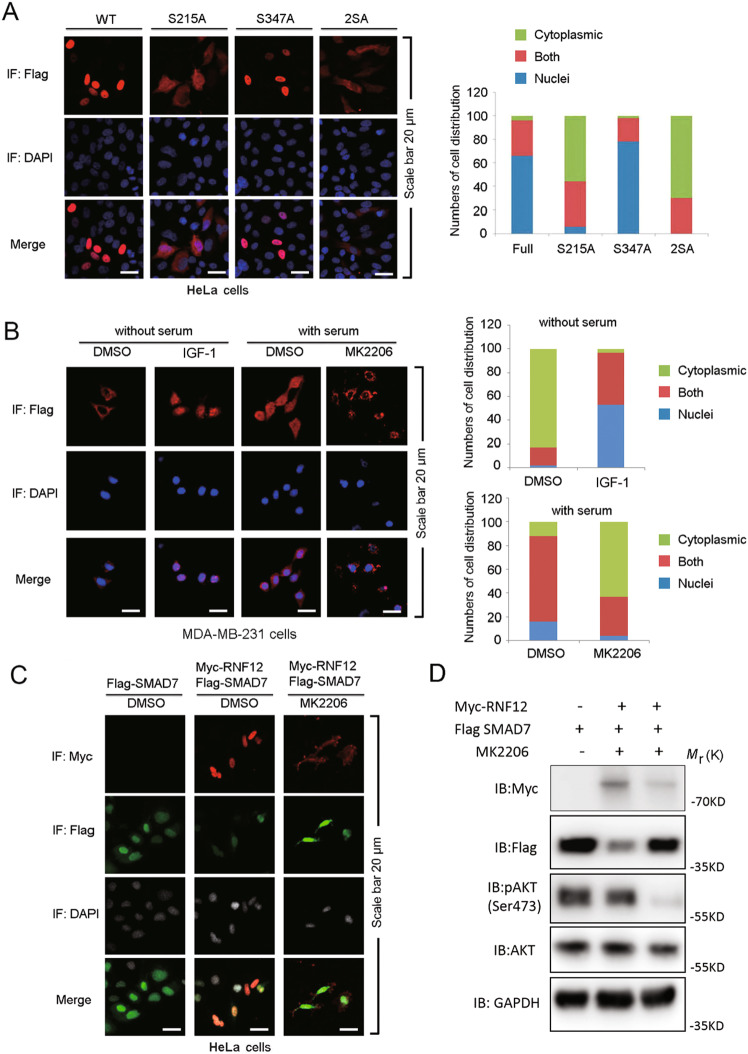
AKT activation promotes the nucleus localization of RNF12. **A** Immunofluorescence and 4, 6-diamidino-2-phenylindole (DAPI) staining of HeLa cells stably transfected with RNF12 WT, S215A, S347A/T349A, or S215A/S347A plasmids. Left panel: Number of cells (A) were analyzed according to the following subgroups: cytoplasm, nuclear, or at both locations (both). **B** Immunofluorescence and DAPI staining of MDA-MB-231 cells treated with IGF-1 (200 ng/mL) and MK2206 (5 μM). **C** Immunofluorescence and DAPI staining of HeLa cells transfected with Flag-RNF12 and GFP-SMAD7, treated with DMSO or MK2206 (5 μM) for 8 h. **D** Western blotting experiments relevant with **C**.

It was reported that AKT played an important role in regulating the nuclear accumulation of its substrates [39]. Therefore, we tested the influence of RNF12 subcellular localization by AKT signaling. Consistent with previous study [28], we found that phosphorylation of Ser215 in RNF12 is needed for its nuclear localization in MDA-MB-231 cells. The RNF12 S215A mutant protein is mainly localized in the cytoplasm. Mutation of serine 347 to alanine (S347A) exerted little effect on RNF12 localization, which, similar to RNF12 WT, remained mainly in the nucleus (Fig. 4A). Moreover, when the subcellular distribution of endogenous RNF12 in MDA-MB-231 cells was analyzed in the absence or presence of AKT activation, it was found that AKT signaling was essential for nuclear localization of RNF12. This result was consistent with those of ectopic expression experiments. Challenging MDA-MB-231 cells with IGF-1 promoted the nuclear localization of RNF12, whereas the selective AKT kinase inhibitor, MK2206 [38], nearly abolished the nuclear localization of RNF12 in cultured cells (Fig. 4B). Next, we explored the effect of AKT-induced changes in the subcellular distribution of RNF12 and its effect on its substrate SMAD7. Inhibitory SMAD7 acts as a substrate for RNF12, which is consistent with a previous report [40], which indicated that when RNF12 and SMAD7 were co-expressed, RNF12 decreased SMAD7 expression levels (Fig. 4C). Under basal growth conditions, SMAD7 is predominantly localized in the nucleus [40]. Notably, we found that inhibition of AKT signaling stabilizes the RNF12 substrate SMAD7 protein. These results were also consistent with the relevant western blotting experiments in vitro (Fig. 4D). Taken together, these findings suggest that nuclear-localized AKT inhibits inhibitory SMAD7 levels and thereby potentiates TGF-β signaling.

### AKT-mediated phosphorylation of RNF12 regulates its function in TGF-β signaling

**Fig. 5:**
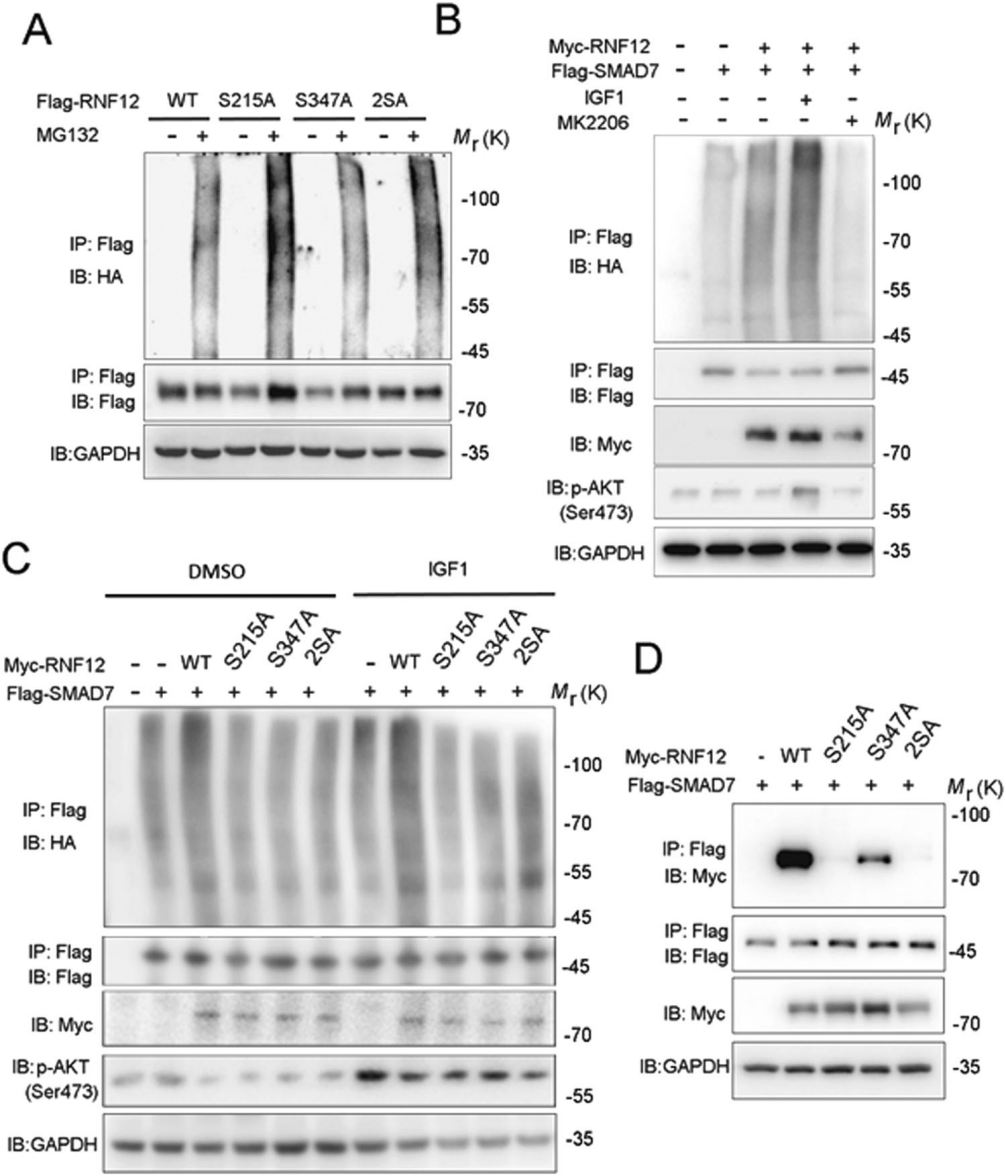
AKT signaling promotes RNF12-mediated SMAD7 ubiquitination. **A** Immunoblot analysis of whole-cell lysate and immunoprecipitations derived from HA-Ub-expressing HEK293T cells transfected with Flag-RNF12 WT, RNF12 S215A, or RNF12 S347A/T349A. **B** AKT signaling regulates RNF12-mediated ubiquitination of SMAD7. Immunoblot analysis of whole-cell lysate and immunoprecipitations derived from HA-Ub-expressing HEK293T cells transfected with Flag-SMAD7 and Myc-RNF12, treated with IGF-1 (200 ng/mL) or MK2206 (5 μM) for 8 h. **C** RNF12 mutations attenuated ubiquitination of SMAD7 by RNF12. Immunoblot analysis of whole-cell lysates and immunoprecipitation derived from HA-Ub-expressing HEK293T cells transfected with Flag-SMAD7 and Myc-RNF12 mutants. **D** RNF12 S215A mutation attenuated interaction between RNF12 and SMAD7. HEK293T cells were co-transfected with Flag-SMAD7 and Myc-RNF12 wild-type or mutant plasmids. At 48 h post-transfection, cell lysates were harvested and subjected to anti-FLAG immunoprecipitation followed by anti-Myc western blotting. Protein expression was confirmed by western blotting using total cell lysates.

RNF12 is not a stable protein and undergoes ubiquitination [41]. RNF12 S215A was degraded faster than RNF12 WT (Fig. 3F). Consistent with this finding, RNF12 S215A demonstrated an increased level of conjugation with polyubiquitin chains in the presence of the proteasome inhibitor, MG132, when compared with RNF12 WT or S347A/T349A (Fig. 5A). These results suggested that S215 was the main site from which AKT signaling regulated RNF12 stability. Impaired protein half-life of RNF12 S215A was likely due to elevated ubiquitylation and proteasomal degradation.

In addition, we examined the effect of AKT on RNF12-mediated SMAD7 protein degradation. We found that knockdown RNF12 in MDA-MB-231 cells increased the protein levels of endogenous SMAD7 (Supplementary Fig. 5A) and RNF12 S215A attenuated the degradation of SMAD7 protein (Supplementary Fig. 5B). Strikingly, the level of SMAD7 ubiquitination mediated by RNF12 was increased when stimulated using IGF-1 (which triggers AKT activation) but decreased when treated with the selective AKT kinase inhibitor, MK2206 (Fig. 5B). Furthermore, both RNF12 S215A and S347A/T349A mutations inhibited SMAD7 ubiquitination (Fig. 5C). The observation that S215A, and not S347A, affects RNF12 protein stability, indicated that the effect of AKT signaling on RNF12-mediated SMAD7 ubiquitination was not solely due to the regulation of RNF12 stability. Thus, we also detected an association between RNF12 phosphorylation site mutations and SMAD7 (Fig. 5D). We found that both S215A and S347A mitigated its association with SMAD7, with RNF12 S215A showing a more striking effect (Fig. 5D). This may be caused by the predominantly cytoplasmic localization of RNF12 S215A and the nuclear localization of SMAD7 in the absence of TGF-β stimulation [40]. We also found the interaction between endogenous RNF12 and SMAD7 proteins was also significantly attenuated by the AKT inhibitor MK2206 (Supplementary Fig. 5C). These results suggested that AKT-mediated RNF12 phosphorylation is required for RNF12 to relocate to the nucleus and interact with SMAD7.

**Fig. 6:**
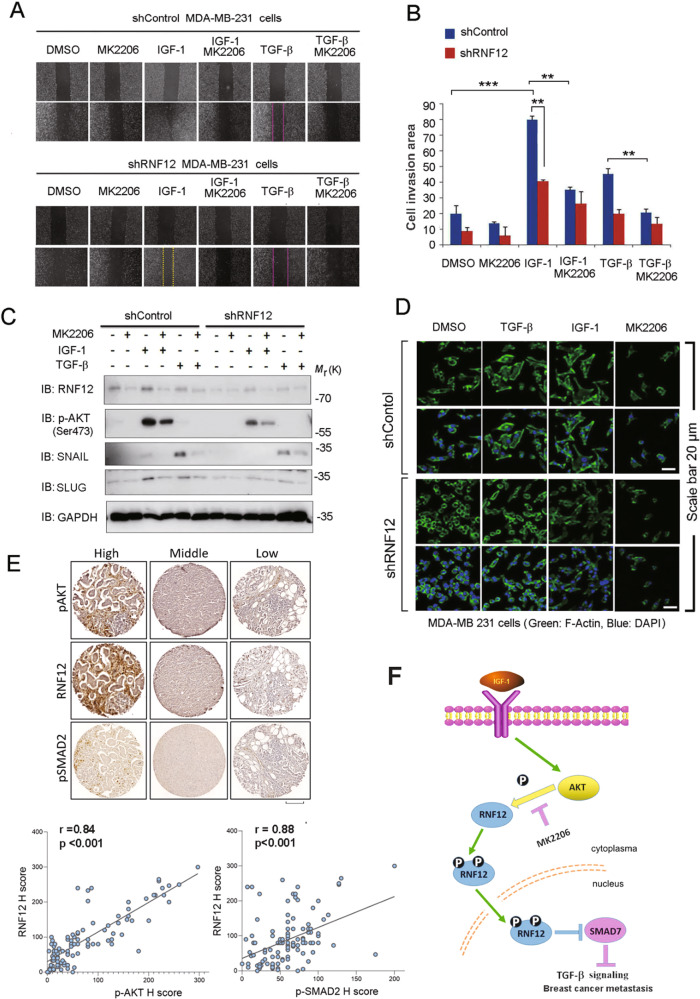
AKT signaling increases TGF-β/RNF12 signaling and related metastasis. **A** Control and RNF12 stably depleted MDA-MB-231 cells were plated for the cell wound-healing assay. Sub-confluent MDA-MB-231 cells were wounded using a 20 µL pipette tip and treated with or without the indicated ligands and reagents for 16 h. The wound-healing area at 16 h after wounding was normalized to that at the start time. **B** Statistical analyses were performed with Student’s *t*-test (*0.01 < *p* < 0.05; **0.001 < *p* < 0.01; ***p < 0.001). For the bar charts, data are plotted as the mean ± SD of at least three independent experiments. **C** Immunoblotting analysis of mesenchymal marker expression treated with IGF-1, TGF-β, and MK2206 as indicated. **D** RNF12 depletion inhibits AKT- and TGF-β-induced EMT in MDA-MB 231 cells. Cells were treated with TGF-β for 24 h. F-actin was stained with phalloidin conjugated with tetramethylrhodamine isothiocyanate (TRITC). **E** Representative images of RNF12 and matched pAKT and pSMAD2 immunohistochemistry results are shown. Graphs in the right panel show the correlation between RNF12 expression levels and those of pSMAD2 in breast cancer TMAs. Spearman’s test was used, and the correlation coefficients and the two-tailed significance values (*p* < 0.001) are shown. Scale bar = 300 μm. **F** Proposed model for the crosstalk between AKT signaling and RNF12 in breast cancer metastasis.

Next, we analyzed the cooperation between AKT and TGF-β in regulating RNF12 and biological responses. TGF-β-induced migration of MDA-MB-231 cells was inhibited by RNF12 knockdown (Fig. 6A). This response was also inhibited by the selective AKT kinase inhibitor, MK2206 (Fig. 6A), which result was in line with previous reports that support a role for AKT signaling in TGF-β-induced migration and epithelial-mesenchymal transition (EMT) of breast cancer cells [8]. To further investigate the role of RNF12 in IGF-1/AKT signaling, we examined the effect of RNF12 depletion on IGF-1 induced migration of MDA-MB-231 cells. We found that depletion of RNF12 reduced IGF-1 induced cell migration (Fig. 6A, B), suggesting that RNF12 plays a positive role in AKT-induced MDA-MB-231 migration. Immunoblotting showed that AKT and TGF-β signaling cooperated with each other to induce the expression of EMT-associated markers such as SLUG and SNAIL, which were decreased in RNF12-depleted cells (Fig. 6C). In addition, RNF12 depletion inhibited TGF-β or insulin-induced morphological phenotypes in MDA-MB 231 cells as measured by filamentous (F-) actin staining using phalloidin (Fig. 6D). This result suggests the possibility of the interplay between AKT signaling and RNF12 in the TGF-β pathway. To further support this, we re-examined the 175 clinical samples obtained from breast cancer patients. Notably, the expression of RNF12 was positively correlated with pAKT and pSMAD2 expression levels (Fig. 6E). All the above results indicate that AKT is important for the phosphorylation and nucleus localization of RNF12 in breast cancer cells, which further upregulate TGF-β signaling and promote breast cancer metastasis (Fig. 6F).

## Discussion

Aberrant AKT activation and the TGF-β pathway appear to cooperate in promoting breast cancer progression. However, the underlying mechanisms of the crosstalk between AKT and TGF-β pathways remain unclear. We found that the E3 ubiquitin ligase, RNF12, plays a pivotal role in the interplay between these two pathways. Our results suggest a model in which AKT activation by IGF induces RNF12 phosphorylation, which triggers its nuclear accumulation and increases its stability. The phosphorylated RNF12 becomes more effective in inducing the ubiquitination and degradation of SMAD7, therefore, enforces TGF-β-induced invasion and metastasis responses in breast cancer cells (Fig. 6F).

We found that RNF12 is highly expressed in invasive breast cancer cells and its high level was associated with poor prognoses of breast cancer patients. We further observed that RNF12 expression was particularly high in lymph node-positive breast cancer cells, which demonstrate a high probability for breast cancer metastasis. Consistent with these findings, RNF12 was expressed at higher levels in more aggressive basal breast cancer cell lines than in luminal ones. In addition, RNF12 promoted breast cancer cell migration, invasion, and experimental metastasis in in vitro and in vivo models. RNF12 is a potent activator of TGF-β signaling [11], which play important role in triggering the invasive and metastatic traits of mesenchymal triple-negative breast cancer cell lines. Consistent with this notion and high RNF12 expression patterns seen in invasive breast cancer, we observed that RNF12 is needed for efficient induction of TGF-β of mesenchymal markers and TGF-β target genes in the malignant phenotypes of MDA-MB 231 cells. These findings hinted at a mechanistic basis for the role of RNF12 in breast cancer cell invasion and metastasis.

Moreover, a strong positive correlation was found between RNF12 and activated pAKT and pSMAD2 in clinical breast cancer samples. This prompted us to consider RNF12 as a possible substrate for AKT. Two potential substrates were identified: Ser215 and S347. We observed that AKT binds and phosphorylates (human) RNF12 at Ser215 and Ser347. Consistent with previous results [27, 28], Ser215 was found to be important for nuclear accumulation. Moreover, it promoted the stability of RNF12 and its ability to ubiquitinate SMAD7. The latter result is in line with a recent report in which SRSFPK was shown to phosphorylate RNF12 at (mouse) Ser214, (and other serine residues) which ensured its delivery to nuclear substrates, such as SMAD7, increasing its E3 ubiquitin ligase activity thereby [27]. Our finding that AKT-induced phosphorylation increased the stability of RNF12, while simultaneously increasing ubiquitination activity was somewhat unexpected. RNF12 auto-ubiquitinates and targets itself for proteasomal degradation. Further studies are required to investigate the link between ubiquitination types to explain these findings. Our results demonstrated that S347A RNF12 phosphorylation at S347 is not involved in the regulation of subcellular distribution of RNF12, and only exerted a minor effect on RNF12 function.

Our findings indicated that RNF12 and AKT cooperated to promote breast cancer cell invasion and metastasis, in part by promoting pro-oncogenic responses to TGF-β. As TGF-β displays both tumor-suppressing and tumor-promoting activities, directly inhibiting TGF-β, via TGF-β receptor kinase inhibitors or antibodies that block ligand-receptor interactions, to treat cancer remains challenging [42]. Therefore, developing new drugs on RNF12 may provide potential avenue to treat breast cancer patients with high RNF12 expression. Furthermore, the combination therapy by targeting AKT, RNF12 and TGF-β in aggressive breast cancer can be studied in future research.

## Materials and methods

### Plasmids and reagents

RNF12 and related expression constructs were cloned and verified via DNA sequencing. RNF12 S215A and RNF12 S347A/T349A were generated by site-directed mutagenesis and confirmed via DNA sequencing. Myr-AKT1 constructs were kindly provided by P. Coffer (University Medical Center, Utrecht, The Netherlands). The reagents used were IGF-1 (R&D 291-G1), MK2206 (Selleck, S1078), MG132 (Selleck, S2619), cycloheximide (CHX) (Sigma, C104450), and SB431542 (Millipore, 616461). The antibodies used for immunoprecipitation (IP), immunoblotting (IB) and immunofluorescence were, c-Myc (IB; no.9402, Cell Signaling), HA (IB; Y-11, sc-805, Santa Cruz), HA (IB; 12CA5, home-made), Flag (IB; M2, Sigma), β-actin (IB; A5441, Sigma), RNF12 (IB, CST) (IP; U0635, Proteintech), AKT (IB, Cell Signaling) (IP; no. 2938, Cell Signaling), phosphor-AKT substrate (RXRXXS*-T*) (IB; no. 10001, Cell Signaling), phosphor-AKT (Ser473) (IB; no. 9271, Cell Signaling), tubulin (IB; no. 2146, Cell Signaling), SMAD2-3(IB; 610842 BD), phospho-SMAD2/3(IB; no. 3101, Cell Signaling), SMAD7(IB; ab216428, Abcam)(IB; MAB2009, R&D), Ub (IB; P4D1, Santa Cruz), SNAIL (IB; C15D3, Cell Signaling), and SLUG(IB; C19G7, Cell Signaling). RNF12 Ser215 phosphorylation-specific antibody was produced in a biotechnology company (GL Biochem Ltd, Shanghai).

### Patient selection

A high-density multiple organ tumor tissue microarray (TMA) with adjacent normal tissue was purchased from US Biomax (MC5002). The breast cancer TMA analyses were performed on TMAs that were constructed from 175 tumor samples from the ORIGO cohort [43]. Patients included in this cohort were diagnosed with a primary breast tumor and treated in the Leiden University Medical Center (LUMC) between 1997 and 2003. Informed consent was obtained from all patients.

### Cell lines

The human kidney epithelial HEK293T cell line and the cervical cancer HeLa cell line, as well as breast cancer MDA-MB-231 cell lines, were originally obtained from the American Type Culture Collection (ATCC). All cell lines were cultured in DMEM supplemented with L-glutamine, 10% fetal bovine serum (FBS), and 1:100 penicillin/streptomycin (Gibco, Invitrogen, Blijswijk, Netherlands). All cell lines were regularly tested to ensure the absence of mycoplasma. In addition, the cell lines were authenticated and verified as free of contamination from other cell lines and microbes.

### Lentiviral transduction and generation of stable cell lines

Lentiviruses were produced by transfecting HEK293T cells with shRNA-targeting plasmids and the helper plasmids, pCMV-VSVG, pMDLg-RRE (gag/pol), and pRSV-REV. Cell supernatants were harvested 48 h after transfection and were used either to infect cells or stored at −80 °C. To obtain stable cell lines, cells were infected at the low confluence (20%) for 24 h with lentiviral supernatants diluted 1:1 with normal culture medium in the presence of 5 ng/mL of polybrene (Sigma). Forty-eight hours following infection, cells were placed under puromycin selection for 1 week and then passaged before use. Puromycin (2 µg/mL) was used to maintain the MDA-MB-231 cell line. Lentiviral shRNAs were obtained from Sigma (MISSION® shRNA). Five shRNAs were identified and tested, and the two most effective shRNAs were used in the experiments. We used TRCN0000004139 (#1) and TRCN0000004142 (#2) to knock down RNF12. The MCF-7 stable cell line with ectopic expression of RNF12 was constructed by pLV-GFP-RNF12 lentivirus (Genechem Co.,Ltd., Shanghai).

### Zebrafish xenograft invasion assay

The transgenic zebrafish line, Tg (*fli1*: GFP), was raised, staged, and maintained according to standard procedures and in compliance with the stipulations of the local Institutional Committee for Animal Welfare of the LUMC. MDA-MB-231 cells were injected into zebrafish embryos, as previously described [8]. Briefly, ~400 mCherry MDA-MB-231 cells were injected into the duct of Cuvier (DoC). After implantation, zebrafish embryos were checked for correct injection, misinjected/non-viable embryos were discarded, and remaining embryos were maintained thereafter at 33 °C. Cells that had extravasated from blood vessels and were present in the avascular tail fin area were analyzed for extravasation [8]. Two independent experiments with at least 30 embryos per group were performed.

### Nude mice and experimental lung metastasis assay

Nude mice were purchased from the Animal Husbandry Centre of the Beijing Institute of Cell Biology, Academia Sinica, Beijing, China. All experiments were approved by and conducted in accordance with the guidelines of the Institutional Animal Care and Use Committee at Peking Union Medical College, Chinese Academy of Medical Science. For lung metastasis formation, 1 × 10^6^ MDA-MB-231 cells stably expressing firefly luciferase were injected into the lateral tail vein of nude mice (*n* = 8 per group) at a volume of 0.1 mL [7]. Bioluminescence images were collected after 21–30 days using a Lumina II imaging system (PerkinElmer), which provides data in a color scale depicting the photon flux (photons per second) emitted from xenografted mice.

### Ubiquitination assay

Cells were washed with phosphate buffered saline (PBS) and lysed in two pellet volumes of RIPA buffer (20 mM NAP, pH 7.4, 150 mM NaCl, 1% Triton, 0.5% sodium deoxycholate, and 1% SDS) supplemented with protease inhibitors and 10 mM N-ethylmaleimide. Lysates were sonicated, boiled at 95 °C for 5 min, diluted in RIPA buffer containing 0.1% SDS, and centrifuged at 4 °C (13.200 rpm for 15 min). The supernatant was incubated with specific antibodies and protein A-Sepharose for 3 h at 4 °C. After extensive washing, bound proteins were eluted with 2 × SDS sample buffer and separated by SDS–PAGE followed by western blotting.

### Immunoblotting and immunoprecipitation

Immunoprecipitation and western blotting were performed as previously described [44]. The following primary antibodies were used; RNF12 (U0635, Proteintech), AKT (2938, Cell Signaling), anti-HA (1583816, Roche), anti-c-Myc (sc-789, Santa Cruz), anti-FLAG (F3165, Sigma), and anti-glyceraldehyde phosphate dehydrogenase (GAPDH) (G8795, Sigma). All secondary antibodies were purchased from Sigma-Aldrich. GAPDH protein levels were used as loading controls.

### Transwell and wound-healing assay

For the Transwell assay, cells were plated in medium with 1% FBS in the upper chamber of a Transwell plate (Corning), while a medium containing 10% FBS was placed in the lower well. After incubation for the indicated time, cells were fixed with methanol for 15 min, stained with crystal violet dye for 15 min, washed, and photographed. Experiments were performed at least thrice. For the wound-healing assay, a confluent cell monolayer in a 12-well plate was wounded by directly scraping the cells with a yellow 20 μL pipette tip and treating with growth factors or inhibitors for the indicated time. Results were analyzed by measuring the migrating wound area and comparing it with the initial wound area. Three independent biological experiments were performed.

### Immunohistochemical staining and evaluation

Primary antibodies against RNF12 (1:100; Abcam 22813), pAKT-Ser473 (1:100; Cell Signaling D7E), and pSMAD2-Ser465/467 (1:100; Millipore AB3849) were used for the immunohistochemical staining of formalin-fixed paraffin-embedded TMAs, according to previously described staining protocols [29]. Quantification of staining was expressed as an H score. The H score was determined by the formula 3 × the percentage of strongly stained cells + 2 × the percentage of moderately stained cells + 1 × the percentage of weakly stained cells, yielding a range of 0–300. Scoring was performed blindly by two independent investigators and taken the median.

### Microscopy and analysis

Fixed cells were imaged using PBS-Tween. Fluorescent image acquisition was performed using a Leica MZ16FA stereo microscope or a Leica SP5 STED confocal microscope. Confocal stacks were processed for maximum intensity projection using the Leica software or Adobe Photoshop CS4 software. Images were adjusted for brightness and contrast using Adobe Photoshop CS4. Overlays were created using Adobe Photoshop CS4.

### Cell viability and proliferation assays

Approximately 3000 cells were placed in 48-well plates and subjected to different treatments. Cells in the selected plates were incubated with 3-(4,5-dimethylthiazol-2-yl)−2,5-diphenyl-2H-tetrazolium bromide (MTT) (5 mg/mL) for 4 h and the cell viability was assessed by measuring absorbance at 490 nm daily using a luminal plate reader (Bio-Rad, Hercules, CA, USA). Experiments were performed at least thrice.

### RNA isolation and real-time quantitative PCR (RT-PCR)

Total RNA was extracted with a NucleoSpin RNA II kit (BIOKE, Leiden, Netherlands) according to the manufacturer’s instructions. A RevertAid First Strand cDNA Synthesis Kit (Thermo Scientific, Leusden, Netherlands) was used for reverse transcriptase (RT)-polymerase chain reaction (PCR). RT-PCR was performed on a CFX connect real-time PCR system (Bio-Rad, Veenendaal,) and analyzed using CFX Manager software (version 2.0; Bio-Rad). PCR oligo sequences are listed (Supplementary Table S1). All samples were analyzed in triplicate and normalized to *GAPDH*.

### Statistical analysis

Statistical analyses were performed using Prism 7 software (GraphPad La Jolla, USA). Results are represented by the mean ± SD. Two-way analysis of variance (ANOVA) followed by the two-tailed Student’s *t*-test was used. Statistical significance was set at *p* < 0.05; *0.01 < *p* < 0.05; **0.001 < *p* < 0.01; ***p < 0.001; ****p < 0.0001.

## Supplementary information

Supplementary Figures

Supplementary Table 1

Reproducibility-checklist

## Data Availability

The authors declare that all relevant data of this study are available from the corresponding author on reasonable request.

## References

[1] Hao Y, Baker D, Ten Dijke P (2019). TGF-β-mediated epithelial-mesenchymal transition and cancer metastasis. Int J Mol Sci.

[2] Batlle E, Massague J (2019). Transforming growth factor-β signaling in immunity and cancer. Immunity.

[3] Miyazono K, Katsuno Y, Koinuma D, Ehata S, Morikawa M (2018). Intracellular and extracellular TGF-beta signaling in cancer: some recent topics. Front Med.

[4] Wrana JL, Attisano L, Wieser R, Ventura F, Massague J (1994). Mechanism of activation of the TGF-beta receptor. Nature.

[5] Hill CS (2016). Transcriptional control by the SMADs. Cold Spring Harb Perspect Biol.

[6] Massague J (2008). TGFbeta in cancer. Cell.

[7] Huang F, Shi Q, Li Y, Xu L, Xu C, Chen F (2018). HER2/EGFR-AKT signaling switches TGFβ from inhibiting cell proliferation to promoting cell migration in breast cancer. Cancer Res.

[8] Drabsch Y, He S, Zhang L, Snaar-Jagalska BE, ten Dijke P (2013). Transforming growth factor-β signalling controls human breast cancer metastasis in a zebrafish xenograft model. Breast Cancer Res.

[9] Conery AR, Cao Y, Thompson EA, Townsend CM, Ko TC, Luo K (2004). Akt interacts directly with Smad3 to regulate the sensitivity to TGF-beta induced apoptosis. Nat Cell Biol.

[10] Remy I, Montmarquette A, Michnick SW (2004). PKB/Akt modulates TGF-beta signalling through a direct interaction with Smad3. Nat Cell Biol.

[11] Zhang L, Huang H, Zhou F, Schimmel J, Pardo CG, Zhang T (2012). RNF12 controls embryonic stem cell fate and morphogenesis in zebrafish embryos by targeting Smad7 for degradation. Mol Cell.

[12] Itoh S, ten Dijke P (2007). Negative regulation of TGF-beta receptor/Smad signal transduction. Curr Opin Cell Biol.

[13] Kavsak P, Rasmussen RK, Causing CG, Bonni S, Zhu H, Thomsen GH (2000). Smad7 binds to Smurf2 to form an E3 ubiquitin ligase that targets the TGF beta receptor for degradation. Mol Cell.

[14] Suzuki C, Murakami G, Fukuchi M, Shimanuki T, Shikauchi Y, Imamura T (2002). Smurf1 regulates the inhibitory activity of Smad7 by targeting Smad7 to the plasma membrane. J Biol Chem.

[15] Zhang Z, Fan Y, Xie F, Zhou H, Jin K, Shao L (2017). Breast cancer metastasis suppressor OTUD1 deubiquitinates SMAD7. Nat Commun.

[16] Koinuma D, Shinozaki M, Komuro A, Goto K, Saitoh M, Hanyu A (2003). Arkadia amplifies TGF-beta superfamily signalling through degradation of Smad7. EMBO J.

[17] Hill CS (2012). Inhibiting the inhibitor: the role of RNF12 in TGF-β superfamily signaling. Mol Cell.

[18] Briones-Orta MA, Levy L, Madsen CD, Das D, Erker Y, Sahai E (2013). Arkadia regulates tumor metastasis by modulation of the TGF-β pathway. Cancer Res.

[19] Bach I, Rodriguez-Esteban C, Carriere C, Bhushan A, Krones A, Rose DW (1999). RLIM inhibits functional activity of LIM homeodomain transcription factors via recruitment of the histone deacetylase complex. Nat Genet.

[20] Ostendorff HP, Bossenz M, Mincheva A, Copeland NG, Gilbert DJ, Jenkins NA (2000). Functional characterization of the gene encoding RLIM, the corepressor of LIM homeodomain factors. Genomics.

[21] Johnsen SA, Gungor C, Prenzel T, Riethdorf S, Riethdorf L, Taniguchi-Ishigaki N (2009). Regulation of estrogen-dependent transcription by the LIM cofactors CLIM and RLIM in breast cancer. Cancer Res.

[22] Her YR, Chung IK (2009). Ubiquitin ligase RLIM modulates telomere length homeostasis through a proteolysis of TRF1. J Biol Chem.

[23] Gao R, Wang L, Cai H, Zhu J, Yu L (2016). E3 ubiquitin ligase RLIM negatively regulates c-Myc transcriptional activity and restrains cell proliferation. PLoS One.

[24] Huang Y, Nie M, Li C, Zhao Y, Li J, Zhou L (2017). RLIM suppresses hepatocellular carcinogenesis by up-regulating p15 and p21. Oncotarget.

[25] Jonkers I, Barakat TS, Achame EM, Monkhorst K, Kenter A, Rentmeester E (2009). RNF12 is an X-Encoded dose-dependent activator of X chromosome inactivation. Cell.

[26] Shin J, Bossenz M, Chung Y, Ma H, Byron M, Taniguchi-Ishigaki N (2010). Maternal Rnf12/RLIM is required for imprinted X-chromosome inactivation in mice. Nature.

[27] Bustos F, Segarra-Fas A, Nardocci G, Cassidy A, Antico O, Davidson L (2020). Functional diversification of SRSF protein kinase to control ubiquitin-dependent neurodevelopmental signaling. Dev Cell.

[28] Jiao B, Taniguchi-Ishigaki N, Gungor C, Peters MA, Chen YW, Riethdorf S (2013). Functional activity of RLIM/Rnf12 is regulated by phosphorylation-dependent nucleocytoplasmic shuttling. Mol Biol Cell.

[29] Zhou F, Drabsch Y, Dekker TJ, de Vinuesa AG, Li Y, Hawinkels LJ (2014). Nuclear receptor NR4A1 promotes breast cancer invasion and metastasis by activating TGF-β signalling. Nat Commun.

[30] Herfs M, Hubert P, Kholod N, Caberg JH, Gilles C, Berx G (2008). Transforming growth factor-beta1-mediated Slug and Snail transcription factor up-regulation reduces the density of Langerhans cells in epithelial metaplasia by affecting E-cadherin expression. Am J Pathol.

[31] Chimal-Monroy J, Diaz de Leon L (1999). Expression of N-cadherin, N-CAM, fibronectin and tenascin is stimulated by TGF-beta1, beta2, beta3 and beta5 during the formation of precartilage condensations. Int J Dev Biol.

[32] Wickert L, Chatain N, Kruschinsky K, Gressner AM (2007). Glucocorticoids activate TGF-beta induced PAI-1 and CTGF expression in rat hepatocytes. Comp Hepatol.

[33] Katoh M, Katoh M (2010). Integrative genomic analyses of CXCR4: transcriptional regulation of CXCR4 based on TGFbeta, nodal, activin signaling and POU5F1, FOXA2, FOXC2, FOXH1, SOX17, and GFI1 transcription factors. Int J Oncol.

[34] Grotendorst GR, Okochi H, Hayashi N (1996). A novel transforming growth factor beta response element controls the expression of the connective tissue growth factor gene. Cell Growth Differ.

[35] Wiercinska E, Naber HP, Pardali E, van der Pluijm G, van Dam H, ten Dijke P (2011). The TGF-β/Smad pathway induces breast cancer cell invasion through the up-regulation of matrix metalloproteinase 2 and 9 in a spheroid invasion model system. Breast Cancer Res Treat.

[36] Ferrari G, Cook BD, Terushkin V, Pintucci G, Mignatti P (2009). Transforming growth factor-beta 1 (TGF-beta1) induces angiogenesis through vascular endothelial growth factor (VEGF)-mediated apoptosis. J Cell Physiol.

[37] Liu S, González-Prieto R, Zhang M, Geurink PP, Kooij R, Iyengar PV (2020). Deubiquitinase activity profiling identifies UCHL1 as a candidate oncoprotein that promotes TGFβ-induced breast bancer metastasis. Clin Cancer Res.

[38] Hirai H, Sootome H, Nakatsuru Y, Miyama K, Taguchi S, Tsujioka K (2010). MK-2206, an allosteric Akt inhibitor, enhances antitumor efficacy by standard chemotherapeutic agents or molecular targeted drugs in vitro and in vivo. Mol cancer therapeutics.

[39] Manning BD, Cantley LC (2007). AKT/PKB signaling: navigating downstream. Cell.

[40] Itoh S, Landstrom M, Hermansson A, Itoh F, Heldin CH, Heldin NE (1998). Transforming growth factor beta1 induces nuclear export of inhibitory Smad7. J Biol Chem.

[41] Ostendorff HP, Peirano RI, Peters MA, Schluter A, Bossenz M, Scheffner M (2002). Ubiquitination-dependent cofactor exchange on LIM homeodomain transcription factors. Nature.

[42] Liu S, Ren J, Ten Dijke P (2021). Targeting TGFβ signal transduction for cancer therapy. Signal Transduct Target Ther.

[43] Out AA, Wasielewski M, Huijts PE, van Minderhout IJ, Houwing-Duistermaat JJ, Tops CM (2012). MUTYH gene variants and breast cancer in a Dutch case-control study. Breast Cancer Res Treat.

[44] Zhang X, Zhang J, Zhang L, van Dam H, ten Dijke P (2013). UBE2O negatively regulates TRAF6-mediated NF-κB activation by inhibiting TRAF6 polyubiquitination. Cell Res.

