# Supplementary material for: RNF12 is regulated by AKT phosphorylation and promotes TGF-β driven breast cancer metastasis
Source: Cell Death Dis. 2022 Jan 10;13(1):44. doi: 10.1038/s41419-021-04493-y (PMC8748510; doi:10.1038/s41419-021-04493-y)
Supplement: Supplementary file 1 — Supplementary Figures [file 41419_2021_4493_MOESM1_ESM.docx]

**Supplementary figures**


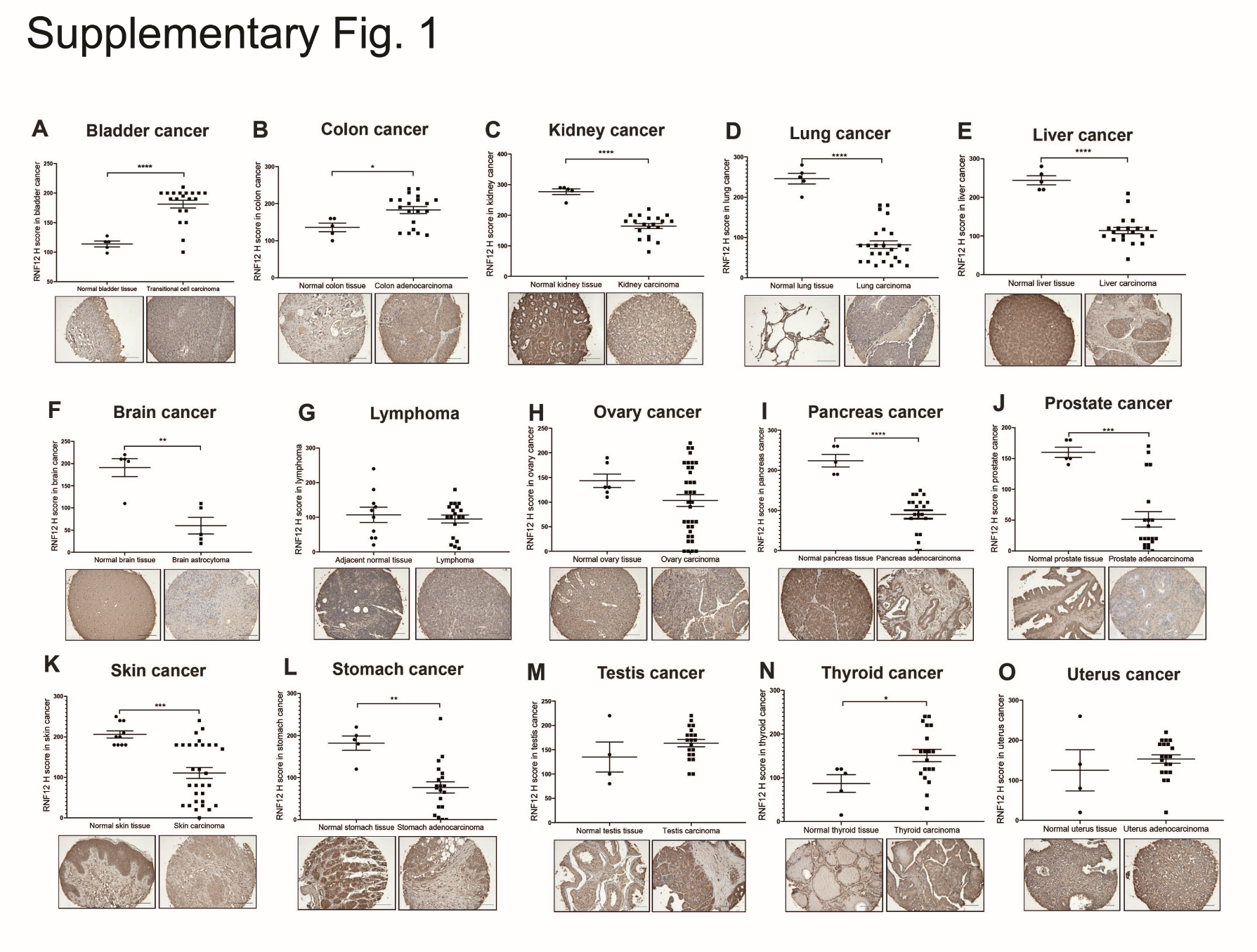


**Supplementary figure 1. Analysis of RNF12 protein levels expressed in specimens of different cancer tissues compared to matched adjacent normal tissues. A.** Bladder cancer. **B.** Colon cancer. **C.** Kidney cancer. **D.** Lung cancer. **E.** Liver cancer. **F.** Brain cancer. **G.** Lymphoma. **H.** Ovarian cancer. **I.** Pancreatic cancer. **J.** Prostate cancer. **K.** Skin cancer. **L.** Stomach cancer. **M.** Testicular cancer. **N.** Thyroid cancer. **O.** Uterine cancer. The upper panel of each figure displays the RNF12 H score statistics in each different cancer type; the bottom panel of each figure consists of representative images of each cancer type and its matched normal tissue. The significance of differences between two independent subjects was determined using the unpaired Student’s t test. P value are indicated by asterisks in the figures: *, P < 0.05, **, P < 0.01, ***, P < 0.001 and ****, P < 0.0001. Scale bar = 300 μm.


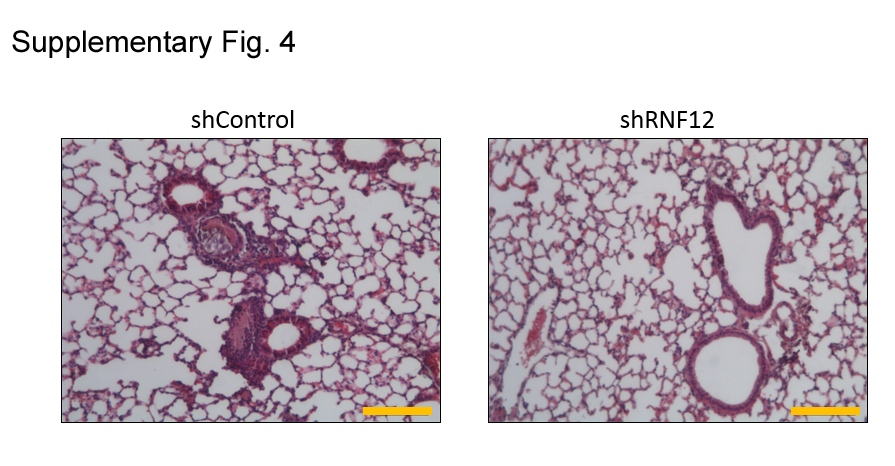


**Supplementary Figure 2.** **Representative Hematoxylin-eosin (HE) staining of the lung colonization of MDA-MB-231 cells in the nude mice.**


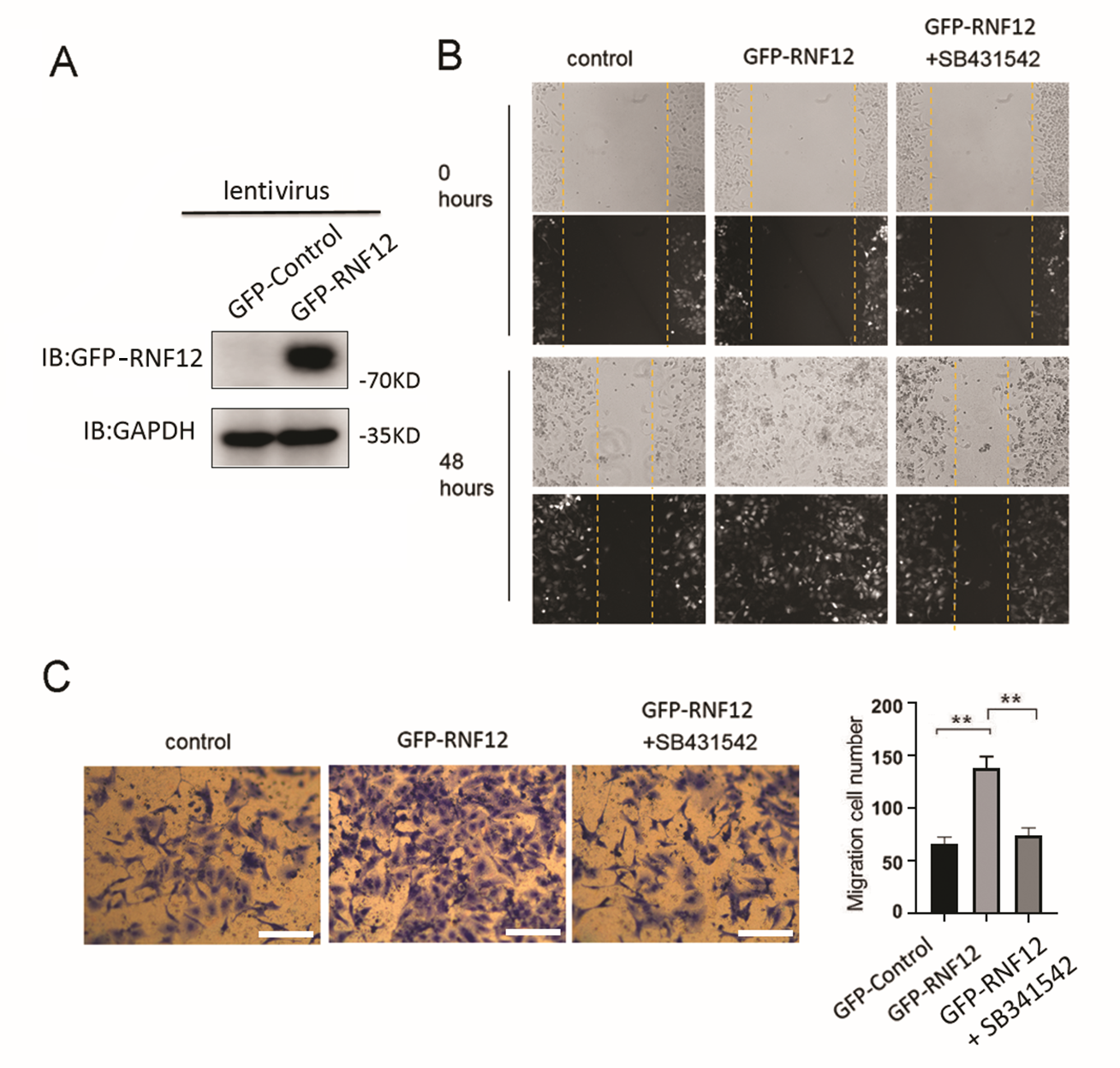


**Supplementary Figure 3.** **Ectopic expression of RNF12 in MCF-7 cells promoted TGF-β related metastasis and migration.**

**A.** Analysis of RNF12 protein levels in MCF-7 stable cell lines with ectopic expression of RNF12. **B.** Control and RNF12 ectopic expressed MCF-7 cells were plated for the cell wound healing assay. Sub-confluent MCF-7 cells were wounded using a 20 mL pipette tip and treated with or without the indicated ligands and reagents for 48 h. **C.** Transwell migration of control or RNF12 ectopic expressed MCF-7 cells. Left panel: Representative images of migrated cells. Right panel, fold changes of migrated cell numbers; mean ±SD of triplicates. Scale bar = 50 μm.


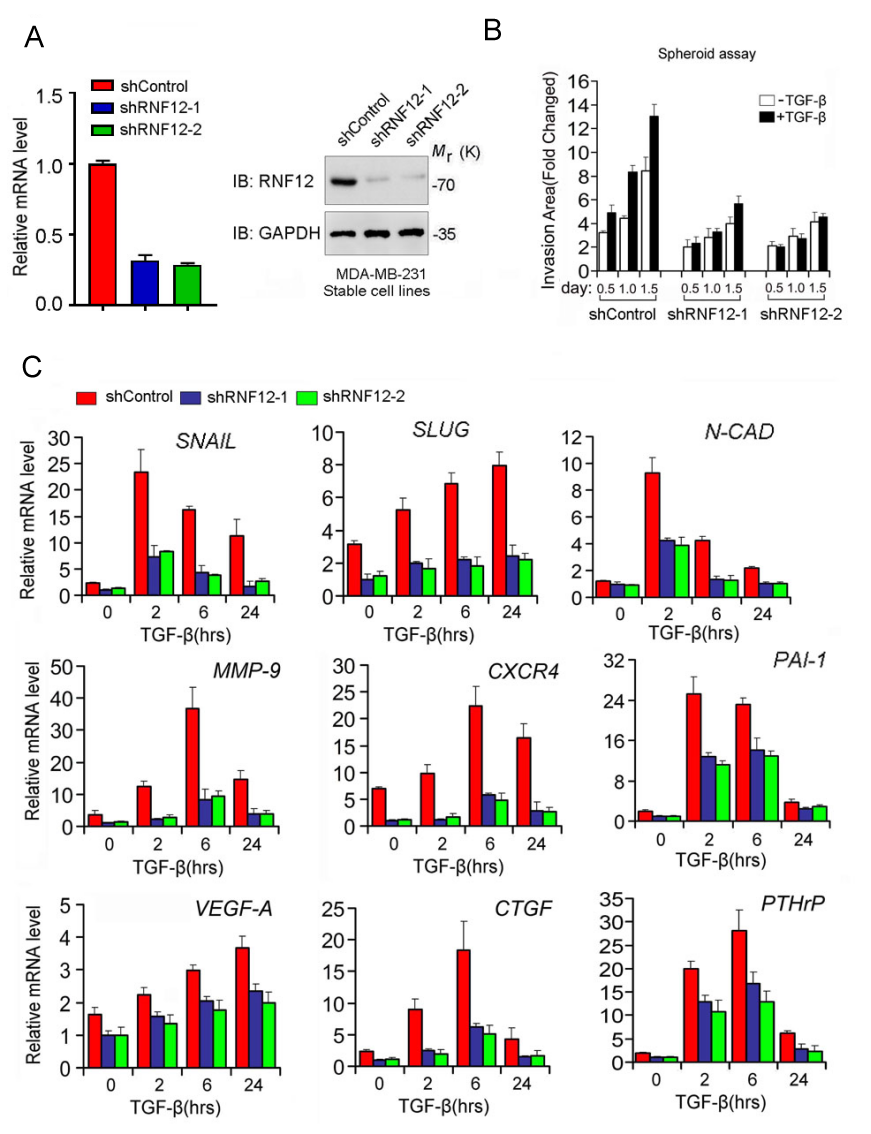


**Supplementary Figure 4. Depletion of RNF12 decreases TGF-β related downstream signaling. A.** qRT-PCR analysis of RNF12 mRNA levels in control and RNF12 depletion MDA-MB-231 stable cell lines. **B.** Spheroid assay of control and RNF12 depletion MDA-MB-231 cell lines. **C.** qRT-PCR analysis of TGF-β target genes *SNAIL, SLUG, N-Cadherin*, *MMP9*, *CXCR4*, *PAI-1*, and *VEGF-A* in control and RNF12 depleted MDA-MB-231 stable cell lines treated with TGF-β (2.5 ng/mL) for 24 h. Values and error bars represent the mean ±SD of triplicates and are representative of at least two independent experiments.


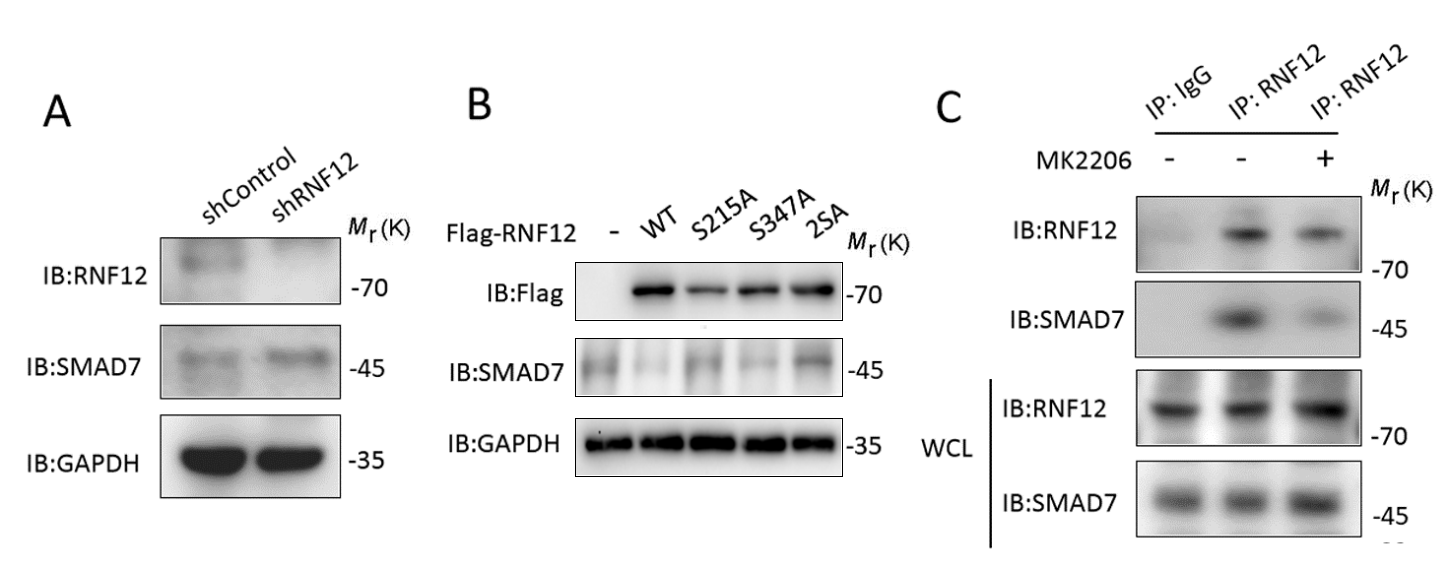


**Supplementary Figure 5.** **AKT signaling regulates RNF12-mediated endogenous SMAD7 protein degradation. A.** Knock-down RNF12 increase SMAD7 protein levels in MDA-MB-231 cells. **B.** Immunoblot analysis of whole cell lysate in the HEK293T cells transfected with Flag-RNF12 WT, RNF12 S215A, or RNF12 S347A. **C.** AKT inhibitor attenuated the interaction between endogenous RNF12 and SMAD7 proteins. MDA-MB-231 cells were treated with DMSO or MK2206 overnight. Cell lysates were harvested and subjected to anti-RNF12 immunoprecipitation followed by anti-SMAD7 western blotting. Protein expression was confirmed by western blotting using total cell lysates.
