# Supplementary material for: RNF12 is regulated by AKT phosphorylation and promotes TGF-β driven breast cancer metastasis
Source: Cell Death Dis. 2022 Jan 10;13(1):44. doi: 10.1038/s41419-021-04493-y (PMC8748510; doi:10.1038/s41419-021-04493-y)
Supplement: Supplementary file 2 — Supplementary Table 1 [file 41419_2021_4493_MOESM2_ESM.docx]

**Supplementary Table 1. Primer list of quantitative PCR**

| **Primer** | **Sequence (5’-3’)** |  |
| --- | --- | --- |
| RNF12 forward | 5'-GCAGCCAACAAGTGAAATTCC-3' |  |
| RNF12 reverse | 5'-CAGGCTCGTTTTCCGAAGAC-3' |  |
| N-cadherin forward | 5’-CAGACCGACCCAAACAGCAAC-3’ |  |
| N-cadherin reverse | 5’-GCAGCAACAGTAAGGACAAACATC-3′ |  |
| SNAIL forward | 5’-CCCCAATCGGAAGCCTAACT-3’ |  |
| SNAIL reverse | 5’-GCTGGAAGGTAAACTCTGGATTAGA-3’ |  |
| SLUG forward | | 5'-ATGAGGAATCTGGCTGCTGT-3' |
| SLUG reverse | | 5'-CAGGAGAAAATGCCTTTGGA-3' |
| MMP9 forward | 5'-TACTGTGCCTTTGAGTCCG-3' |  |
| MMP9 reverse | 5'-TTGTCGGCGATAAGGAAG-3' |  |
| CXCR4 forward | 5'-CAGTGGCCGACCTCCTCTT-3' |  |
| CXCR4 reverse | 5'-CAGTTTGCCACGGCATCA-3' |  |
| pAI-1 forward | 5' CACAAATCAGACGGCAGCACT-3' |  |
| pAI-1 reverse | 5'-CATCGGGCGTGGTGAACTC-3' |  |
| VEGF-A forward | 5'-TACCTCCACCATGCCAAGTG-3' |  |
| VEGF-A reverse | 5'-ATGATTCTGCCCTCCTCCTTC-3' |  |
| CTGF forward | 5'-TGCGAAGCTGACCTGGAAGAGAA-3' |  |
| CTGF reverse | 5'-AGCTCGGTATGTCTTCATGCTGGT-3' |  |
| PTHrP forward | 5'-TTCTTCCCAGGTGTCTTGAG-3' |  |
| PTHrP reverse | 5'-TTTACGGCGACGATTCTTCC-3' |  |
| GAPDH forward | 5'-AACTTTGGCATTGTGGAAGG-3' |  |
| GAPDH reverse | 5'-ACACATTGGGGGTAGGAACA-3' |  |
